# Epidemiology of musculoskeletal disorders among caregivers in nursing homes may depend not only on workload but also on comorbidities

**DOI:** 10.1590/1806-9282.20250723

**Published:** 2025-10-17

**Authors:** Fulvio Alexandre Scorza, Carla Alexandra Scorza, Josef Finsterer

**Affiliations:** 1 Universidade Federal de São Paulo, Escola Paulista de Medicina – São Paulo (SP), Brazil.; 2 Neurology and Neurophysiology Center, Department of Neurology – Vienna, Austria.

Dear Editor,

We read with interest the article by Minghelli et al., which reports on a cross-sectional study investigating the epidemiology of work-related musculoskeletal disorders (WMSDs). The study, conducted using a questionnaire, involved 251 formal caregivers aged 21–65 years working in a nursing home, all of whom had 1–3 WMSDs in the last 12 months^1^. The most common types of WMSD were low back pain (27%), non-specific pain (22%), and tendinopathy (21%) in the lumbar spine, shoulder, or cervical spine^1^. The predominant triggers of the complaints were dislocations or repetitive movements^1^. It was concluded that WMSDs are prevalent among nurses, which calls for the development of appropriate prevention programs^1^. The study is noteworthy, but several points should be discussed.

The first point is that the included patients are not screened for depressive symptoms^1^. Since pain can be a manifestation of depression and vice versa^2^, it is conceivable that at least some of those who reported musculoskeletal pain were actually experiencing depression due to work-related conditions (e.g., burnout, stress, and night shifts) or personal circumstances (e.g., chronic relationship conflict, death of a loved one, and a history of depression). The most common scales used to assess depressive symptoms include the Beck Depression Inventory, the Hamilton Depression Rating Scale, and the Montgomery–Asberg Depression Rating Scale^3^.

The second point is that comorbidities are not included in the analysis^1^. As the age range was from 21 to 65 years, it is conceivable that at least some of the included patients had a comorbidity that either explained or exacerbated the symptoms. Particularly in older nurses (121 had a career of more than 6 years), it is possible that comorbidities contributed to the reported clinical picture. Low back pain can also be caused by viral infections, fever, arthritis, ankylosing spondylitis, osteoporosis, or smoking (coughing, which can lead to disc herniation). Additional causes of neck pain include cervical dystonia, cervical spondylosis, fibromyalgia or hernia, myofascial pain syndrome, rheumatoid arthritis, spinal stenosis, tension-type headaches, or a previous whiplash injury^4^. Have all these alternative causes of low back and cervical pain been ruled out?

The third point is that the definition of WMSDs does not take into account that WMSDs can be processed individually by sufferers, depending on their pain threshold, available pain-processing mechanisms, personality type, psychosocial context, current medication and attitudes to work, society, and symptoms^5^. It is therefore possible that not all of the patients included will take a day off or be able to continue working. Those who do not take sick leave or change jobs may not be captured by the study design, and the number of people with WMSDs may in fact be much higher than those who meet the specified inclusion criteria.

The fourth point is that there is a discrepancy between the inclusion criteria^1^. On the one hand, caregivers who have been working for at least 3 months were included; on the other hand, those who have had WMSDs within the last 12 months were included. Those working for less than 12 months could not fulfil the other inclusion criterion.

In summary, this interesting study has limitations that put the results and their interpretation into perspective. Addressing these limitations could strengthen the conclusions and corroborate the study's message. As the causes of WMSDs are more diverse than assumed and individual treatment can vary greatly from patient to patient, the results should be interpreted with caution. However, it remains undisputed that patients with WMSDs need to be identified and provided with appropriate treatment.

## Data Availability

The datasets generated and/or analyzed during the current study are available from the corresponding author upon reasonable request.
